# Versatile IEEE-488 data acquisition and control routines for a diode array spectrophotometer

**DOI:** 10.1155/S1463924691000160

**Published:** 1991

**Authors:** Paul M. Shiundu, Adrian P. Wade

**Affiliations:** Laboratory for Automated Chemical Analysis Chemistry Department University of British Columbia 2036 Main Mall. Vancouver British Columbia V6T 1Y6 Canada

## Abstract

The UV-visible diode array spectrophotometer is a work-horse instrument for many laboratories. This article provides simple data acquisition and control routines in Microsoft QuickBasic for a HP-8452A diode array spectrophotometer interfaced to an IBM PC/XT/AT, or compatible, microcomputer. These allow capture of full spectra and measure absorbance at one or several wavelengths at preset time intervals. The variance in absorbance at each wavelength is available as an option.


Journal of Automatic Chemistry, Vol. 13, No. 3 (May–June 1991), pp. 83–92

### Versatile IEEE-488 data acquisition and control routines for a diode array spectrophotometer†

**Paul M. Shiundu and Adrian P. Wade***

*Laboratory for Automated Chemical Analysis, Chemistry Department, University of British Columbia, 2036 Main Mall, Vancouver, British Columbia, V6T 1Y6, Canada*

*The UV-visible diode array spectrophotometer is a work-horse instrument for many laboratories. This article provides simple data acquisition and control routines in Microsoft QuickBasic for a HP-8452A diode array spectrophotometer interfaced to an IBM PC/XT/AT, or compatible, microcomputer. These allow capture of full spectra and measure absorbance at one or several wavelengths at preset time intervals. The variance in absorbance at each wavelength is available as an option.*

### Introduction

The diode array spectrophotometer has become an integral part of quantitative and kinetic UV-visible absorbance studies in this laboratory [1,2] and in many others. While operating software purchased from an instrument manufacturer is well suited to most routine analytical needs, it is not flexible enough for some research purposes. Indeed, the nature of research is such that one cannot reasonably expect instrument companies to be able to foresee all needs in advance. Therefore, there is a need for the researcher to be able to write specialized instrument control and data acquisition routines to meet specific research purposes. An example is integration of spectrometer into a larger automated analytical instrument [3].

The manufacturer of the diode array spectrophotometer used here (a Hewlett-Packard Model 8452A) provides, as an option, a library of command routines which programmers can incorporate into their own code. Languages supported include Hewlett-Packard (HP) series 200 PASCAL and BASIC, IBM BASICA and Microsoft QuickBasic. The task of programming versatile or specialist control and data acquisition routines, however, is still not straightforward. In this short article, the HP-8452A hardware is discussed and some general routines written at a higher level in Microsoft QuickBasic‡ are presented. These initialize the spectrophotometer, execute control functions, and acquire data. These routines have been integrated into stopped flow kinetics programs and general methods development software for flow injection analysis. The routines may readily be adapted for other uses of the spectrophotometer and the defaults changed. They could also be adapted for use with other HP-IB compatible devices, such as meters and transient digitizers.

The HP 8452A diode array is a single-beam microprocessor-controlled UV-visible spectrophotometer, which provides 316 diodes across the wavelength range 190–820 nm at 2 nm resolution. It can be controlled by an IBM PC/XT/AT or compatible computer using HP's routines. The spectrophotometer has two communications interfaces: a parallel Hewlett-Packard Interface Bus (HP-IB) and a serial HP-IL interface. Most commonly used is the HP-IB: this IEEE-488 type, high-speed, general purpose, digital interface provides a simple means to control and acquire data from up to 15 instruments or devices, and requires a single adapter card in the control computer. Clearly defined functions exist for each of the 24 IEEE-488 lines: eight are lines for data transmission; another eight for handshaking, and the rest for grounding and shielding. Total transmission pathlengths over the interconnecting cables cannot exceed 20 m without additional buffering and the length of cable per device is limited to 2 m. This design provides a maximum data transfer rate of 1 Mbyte/s, with minimal system cost and complexity. This article assumes that instrument-computer communications will be across an HP-IB interface, using the routines found in the HP-IB Command Library.

The diode array spectrophotometer has become an analytical workhorse in this laboratory. Applications fall into the following categories:

*Scanning*: In the general scan mode, the spectrophotometer can acquire and display the absorption spectrum (absorbance versus wavelength) of a sample over the entire wavelength range, over any user-specified portion of this range, or at up to six individual wavelengths. Transmittance or absorbance measurements are possible. Spectra may be as intensities (normal mode) or as derivative spectra. In each case, it is possible to acquire and store the variance of the values recorded at each wavelength. This provides greater confidence in the values obtained.

*Quantitation*: The concentration of an unknown may be determined by its absorbance relative to that from known standards. Perhaps 10 standard solutions are run to yield a calibration curve. A model equation is then obtained using a standard linear or second order least squares fit.

*Kinetics*: Time based scans on reaction mixtures allow calculation of rate constants. This can be done

* Author to whom correspondence should be addressed.
† The authors would appreciate hearing from scientists who find this article useful.
‡ These routines are placed in the public domain, and may be used freely, but may not themselves be sold for profit nor included in a larger work to be sold for profit without written authorization from the authors of this paper.

0142–0453/91 $3.00 © 1991 Taylor & Francis Ltd.

repeatedly for up to six individual wavelengths in as little as 0·1 s intervals. Alternatively, a wavelength range may be specified by the user. Data can be displayed in either graphical or tabular form, in real time. Use of different temperatures via a thermostated cell allows calculation of activation energies.

### Hardware and software requirements

(1) HP 8452A Diode Array Spectrophotometer and HP 82990A HP-IB Interface and Command Library for MS-DOS.
(2) IBM PC/XT/AT/386 compatible microcomputer with HP-IB interface card.
(3) Microsoft QuickBasic v. 4·00 or 4·5, with MS-DOS version 3·30 or higher.

### Description of the routines provided

The main module, where all user defined subprograms, HP-IB interface library subprograms, HP-IB driver variables and common shared variables are declared, is called HPCONTRL.BAS. The HP-IB subprograms must be present as a QuickLibrary, which is invoked at the time of execution by the command:

QB HPCONTROL/L HPIB.QLB

or may be compiled along with the routines presented here by the commands:

BC HPCONTROL.BAS

LINK HPCONTROL.OBJ + HPIB.OBJ,,,,

Constants such as the device address (HPADD&), HP-IB select code (ISC&), diode array status flag, and single wavelength detection flag are defined here and code necessary to access the command library subroutines and HP-IB drivers is included. It also outputs the banners and instructions to the user on the screen.

Initialization of the diode array spectrophotometer is achieved by the user-defined subroutine HPINIT. Parameters for absorbance measurements such as integration time and sampling intervals are defined through the PARINIT subroutine. The user may choose to monitor single, multiple, or a range of diodes. Options to have the shutter closed or open between repeated measurement cycles and/or to calculate the variance of absorbance values are provided, but are constrained by integration and sampling time intervals. Since shutter processing takes *ca.* 0·5 s, the sampling interval (time between repeat measurements) must be greater than the integration time + 0·5 s, or a run time error will result. When variance data are required, the time taken by the instrument to calculate these must also be considered. Using these routines, a sampling interval of as little as 0·6 s is possible for a full spectrum without variance data, and 1·8 s is needed per full spectrum if variance data is taken at every diode. The length (in bytes) of each record transferred across the HP-IB is dependent on whether variance information is calculated, and is given by:

*# of diodes * (data point size + variance data size) + header size + endline bytes*

Default initial measurement parameters are set as follows:

(1) Use the wavelength range 190–820 m.

(2) Acquire single spectrum with 0·5 s integration time.

(3) Turn lamp on.

(4) Keep shutter closed between measurements.

(5) Set the trigger to be inactive.

(6) Calculate variance data.

(7) Store absorbance readings as binary data.

(8) Use 0·5 s reference integration time with no data output.

The HPINIT subprogram initializes the HP-IB interface and the HP 8452A spectrophotometer by first clearing the device and the HPIB board, and then ascertaining that the 'device identity' specified is correct. It allows the user to enter alternative measurement parameters and contains routines that inform the user of detected error types and wait for appropriate adjustment to be made before continuing.

HPSEND sends the command string required to select a wavelength, sampling interval, data type (absorbance, transmittance), data format (here as BINARY). It may also request the status of the device (for example ready for measure). The command is passed via the HP-IB driver subroutine, IOOUTPUTS. Should an error be detected, a message is printed but execution continues. After the times and wavelengths are set via the HPSEND subroutine, the status of the device is checked for 'ready to measure'. If the spectrophotometer is not ready a reference spectrum is obtained and the status is rechecked. If, after this, it is still not ready, a fatal error results and program aborts.

DETSTRT initiates multiple or single wavelength data acquisition with a 0·1 s integration time and a 0·2 s sampling interval (or 0·6 s sampling frequency for a minimum of 36 diodes) of the spectrophotometer. Absorbance (and optionally variance) data are returned in binary format. It assumes the analytical wavelength(s) and the status (i.e. not initialized, initialized, or initialized and reference taken) of the detector are SHARED COMMON variables.

DETSTOP stops a current measurement cycle and clears the input, output, and error buffers (via the HP-IB driver subroutine IOCLEAR). HPGET returns a data string from the HP diode array through the HP-IB interface via the IOENTERS driver subroutine. If it encounters an error while reading string from device, it prints an error message and re-tries the instruction. If an error is again detected, the routine aborts. GETAVG obtains the data points from the diode array spectrometer via the IOENTERS subroutine. It calls IOENTERS until the length of data string received equals the maximum spaces set (i.e. six bytes for the header, plus three bytes per diode for absorbance/transmittance measurements, and optionally two additional bytes per diode for variance data).

The subroutines HPINIT and DETSTRT must be called first, in that order, before GETAVG can operate. Data points are saved as an array or as single values, for multiple or single diode measurements respectively. WTKEY waits for a key to be pressed. PAUSE waits for a specified delay time before program execution continues. FLSAVE saves the data collected by GETAVG to disk. For multiple wavelength data, the number of diodes and minimum and maximum wavelengths are stored in the file header. These are then followed with an array of data points for every diode, in ascending numerical wavelength order. For single wavelength data, the wavelength used followed by its corresponding data point is stored for every cycle.

### Acknowledgements

The authors acknowledge support from the Natural Science and Engineering Research Council of Canada (NSERC) operating grant 5-80246 and UBC-NSERC grant 5-80085. PMS acknowledges support from a University of British Columbia University Graduate Fellowship.

### References

1. Shiundu, P. M., Wentzell, P. D. and Wade, A. P. *Talanta*, **37** (1990), 329–336.
2. Wade, A. P., Shiundu, P. M. and Wentzell, P. D. *Analytica Chimica Acta*, **237** (1990), 361–379.
3. Wentzell, P. D., Wade, A. P., Shiundu, P. M., Ree, R. M., Hatton, M., Sly, T. J. and Betteridge, D. *Journal of Automatic Chemistry*, **11** (1989), 227–235.

### PROGRAM LISTING

```
'Program: HPCONTRL.BAS, Written by Paul. M. Shiundu and Adrian. P. Wade.
'Laboratory of Automated Chemical Analysis, Univ. of British Columbia,
'Chemistry Department, Vancouver, B.C., Canada., V6T 1Y6.
'=============================================================================
'This program is used to acquire data and control the HP 8452A diode array
'spectrophotometer for both single and multiple wavelengths.  Up to 35 diodes
'can be monitored with data acquisition achieved at least 0.1 s intervals.
'It is also possible to monitor absorbance at all 316 diodes (from 190 to 820 nm),
'at a sampling interval of 0.6 s, or at minimum sampling interval of 1.8 s if
'variance measurements are required.  A QuickLibrary which contains the HPIB drivers
'must be used.  The resolution for this detector is ca. 2 nm."
'*****************************************************************************
'Declare user defined sub-programs and functions.
DECLARE SUB PAUSE (DLYTIM!)
DECLARE SUB HPSEND (CMD$)
DECLARE SUB HPGET (RESP$)
DECLARE SUB FLSAVE (YDATA!(), YVAR(), NDIODE%, DIODFLG%, MINWV%, MAXWV%, WV1%, CNTCHK%)
DECLARE SUB GETAVG (DIODFLG%, WV1%, MINWV%, MAXWV%, CNTCHK%, NLIST%)
DECLARE SUB DETSTRT (DIODFLG%, WV1%, MINWV%, MAXWV%, CNTCHK%, NLIST%, WAVE%())
DECLARE SUB DETSTOP ()
DECLARE SUB HPINIT (NLIST%, WAVE%())
DECLARE SUB PARINIT (NLIST%, WAVE%())
DECLARE SUB WTKEY ()
'Declare all HPIB interface library sub-programs used.
DECLARE SUB IOABORT (ISC&)
DECLARE SUB IOCLEAR (ISC&)
DECLARE SUB IOENTERS (HPADD&, RSP$, MAX.LENGTH%, ACT.LENGTH%)
DECLARE SUB IOOUTPUTS (HPADD&, CMD$, CMDLEN%)
DECLARE SUB IORESET (ISC&)
DECLARE SUB IOSPOLL (HPADD&, STAT%)
DECLARE SUB IOTIMEOUT (ISC&, TIMEOUT!)
'Declare common HPIB driver variables and others variables.
COMMON PCIB.BASERR, PCIB.ERR, PCIB.ERR$, PCIB.NAME$, PCIB.GLBERR
COMMON FALSE%, TRUE%, NOERR, EUNKNOWN, ESEL, ERANGE, ETIME, ECTRL, EPASS
COMMON ENUM, EADDR
COMMON SHARED ISC&, HPADD&, HPSTAT%, SMINT, INTGTM, VRNFLG%, WAVE%()
COMMON SHARED DIODFLG%, NDIODE%, MINWV%, MAXWV%
'=============================================================
'Code necessary for HPIB drivers (as in HP's QB4SETUP.BAS).
        PCIB.ERR = 0
        PCIB.ERR$ = STRING$(64, 32)
        PCIB.NAME$ = STRING$(16, 32)
        PCIB.GLBERR = 0
        CALL DEFERR(PCIB.ERR, PCIB.ERR$, PCIB.NAME$, PCIB.GLBERR)
        PCIB.BASERR = 255
'=============================================================
'Now initialize variables.
        ISC& = 7                    'HPIB select code
        HPADD& = 718                'Device address for diode array
        HPSTAT% = 0                 'Diode array status - not init.
        DETSCL = 1000               'Detector scale factor for the HP
        DIODFLG% = 0                'Assume single wavelength detection
        CLS                         'Now print banners and instructions for user.
        LOCATE 3, 30 : PRINT "HPCONTRL version 1"
        LOCATE 5, 10 : PRINT "HP DATA ACQUISITION FOR SINGLE and MULTIPLE WAVELENGTH PROGRAM"
        LOCATE 8, 18 : PRINT "Laboratory for Automated Chemical Analysis"
        LOCATE 9, 9 : PRINT "Chemistry Department, University of British Columbia, Canada."
        LOCATE 11, 10 : PRINT "========================================================="
        LOCATE 13,1 : PRINT "     This program allows you to acquire data from the
        PRINT "     spectrophotometer for both single and multiple wavelengths."
        PRINT "     The wavelength range available is 190 - 820 nm.  Up to 30 diodes"
        PRINT "     can be monitored with a sampling interval of at least 0.2 sec."
        PRINT "     For more than 30 diodes the minimum sampling interval is 0.6 sec."
        PRINT "     The maximum number of diodes that can be used is 316."
        PRINT : PRINT : PRINT TAB(25);
        CALL WTKEY                  'Wait for a key to be hit
        CLS                         'Clear screen
        CALL PARINIT(NLIST%, WAVE%())   'Initialize parameters for use with diode array
        CALL HPINIT(NLIST%, WAVE%())    'Initialize the diode array spectrophotometer
        CALL DETSTOP                'End data acquisition
        CLOSE #1
END
```

```
SUB DETSTOP
'This subroutine stops the present diode array measurement cycle
'and clears the instrument.  No variables are passed to this subroutine
'directly.  It requires that the interface select code (ISC&)
'and the diode array address (HPADD&) be stored in shared common.
'=====================================================================
'Clear the instrument.
      CALL IOCLEAR(HPADD&)        'Subprogram present in HP-IB library
'Return instrument to a known state i.e. clear all input, output, and
'error buffers and invoke an abort function using "ABT" string command.
      CALL HPSEND("ABT")
END SUB

SUB DETSTRT (DIODFLG%, WV1%, MINWV%, MAXWV%, CNTCHK%, NLIST%, WAVE%())

'This subroutine starts the measurements.  No parameters are passed,

'but the following variables are assumed to be shared common:

'   WV1%     - the wavelength being used for the diode array.

'   MINWV%   - minimum wavelength for multiwavelength studies.

'   MAXWV%   - maximum wavelength for multiwavelength studies.

'   HPSTAT%  - Status of HP diode array (0=not initialized, 1=initialized,

'              2=initialized and reference taken).

'It initiates the process with a user-defined integration time (INTGTM)

'and sampling interval (SMINT).  Both single and multiple wavelength

'options are available.  Data are returned in binary format with or

'without variance.  The wavelength(s) used is(are) stored in the common

'variable WV1% (MNWV1% OR MXWV1%) and must be properly initialized.

'After the time and wavelength are set, the status of the diode array is

'checked to see if it is ready to measure.  If it is not, a reference is run

'and the status is checked again.  If still not ready, a fatal error results.

'==========================================================================

      SELECT CASE DIODFLG%

         CASE 0

            A$ = "WAV1," + STR$(WV1%)          'Set wavelength(s) as string

         CASE 1

            A$ = "WAV0," + STR$(MINWV%) + "," + STR$(MAXWV%)

         CASE 2

            TEMP$ = ""

            FOR I% = 1 TO (NLIST% - 1)

               TEMP$ = TEMP$ + STR$(WAVE%(I%)) + ","

            NEXT I%

            TEMP$ = TEMP$ + STR$(WAVE%(NLIST%))

            A$ = "WAV0," + TEMP$

      END SELECT

      CALL HPSEND(A$)

      TEMP$ = "TIM" + STR$(INTGTM) + ","

      CALL HPSEND("TIM0.1," + STR$(SMINT) + ",20000,0") 'Set time interval (0.2 sec)

      CALL HPSEND("INT0;FMT0,0;VRN0")       'Absorbance, binary, no var

HPSTA: CALL HPSEND("STA")                   'Request status

      CALL HPGET(STA$)

      STAT% = VAL(STA$) AND &H10            'Check ready for measure

      IF STAT% = 0 THEN

         PRINT "Diode array not ready for measurements - waiting."

         CALL PAUSE(2)

         GOTO HPSTA

      END IF

      IF HPSTAT% <> 2 THEN                  'Check for reference

         CALL HPSEND("REF0.5,0")

         CALL PAUSE(1)

         HPSTAT% = 2                        'HP status = reference run

      END IF

      CALL HPSEND("MES")                    'Now ready for measurement.

END SUB

```

```

SUB FLSAVE (YDATA(), YVAR(), NDIODE%, DIODFLG%, MINWV%, MAXWV%, WV1%, CNTCHK%) STATIC

'This subroutine saves the data points

'    YDATA()  - Array for storage of acquired data points.

'    YVAR()   - Array for storage of variance at each diode.

'    NDIODE%  - #. diodes used.

'    DIODFLG% - Flag for single (0) or multiple (1) or list (2) of diodes.

'    MINWV%   - Minimum wavelength.

'    MAXWV%   - Maximum wavelength.

'==========================================================================

      IF DIODFLG% <> 0 THEN               'If multiple diodes, then store data in

         IF CNTCHK% = 0 THEN              'format below

            PRINT #1, NDIODE%;            'Store number of diodes monitored

            PRINT ","; MINWV%;            'Store minimum wavelength

            PRINT ","; MAXWV%             'Store maximum wavelength

            PRINT #1, SMINT               'Store sampling interval

            IF VRNFLG% <> 0 THEN PRINT #1, "Variance data included"  'Include variance.

         END IF

'Store data for every diode from minimum to maximum diode.

         FOR I% = 1 TO NDIODE% - 1

            PRINT #1, YDATA(I%); ",";

            IF VRNFLG% <> 0 THEN PRINT #1, YVAR(I%); ",";   'Store variance values

         NEXT I%

         PRINT #1, YDATA(NDIODE%)

         IF VRNFLG% <> 0 THEN PRINT #1, YVAR(NDIODE%)

      ELSE

         IF CNTCHK% = 0 THEN PRINT #1, WV1%   'Store single value for single diode.

         PRINT #1, YDATA(1)

      END IF

END SUB

SUB GETAVG (DIODFLG%, WV1%, MINWV%, MAXWV%, CNTCHK%, NLIST%)

'This subroutine acquires data points from the spectrophotometer.  The points

'are acquired every SMINT sec if this subroutine is called repetitively.

'The subroutine DETSTRT must be called to begin a measurement cycle

'before this subroutine is called.  (HPINIT must also be called once

'before DETSTRT).  Data points are acquired as they become available.

'========================================================================

      DIM YDATA(NDIODE%)           'Array for response at each diode

      DIM YVAR(NDIODE%)            'Array for response variance at each diode

      NMRES% = 2                   '2 nm resolution per diode.

      ZEROS$ = CHR$(0) + CHR$(0)   'CVL needs 4 byte records (see below).

      HEADER% = 6                  'no. of bytes in header

      BPRD% = 3                    'each diode takes 3 bytes without variance

      NDIODE% = 1                  'flag for single diode monitoring.

'Calculate number of diodes.

      IF DIODFLG% = 1 THEN NDIODE% = ((MAXWV% - MINWV%) / NMRES%) + 1

'Calculate number of spaces for data if variance info. is required.

'Two extra bytes are required for variance corresponding to every diode.

      IF VRNFLG% <> 0 THEN

         MAXSPC% = HEADER% + 5 * NDIODE% 'Max. spaces.

      ELSE

         MAXSPC% = HEADER% + 3 * NDIODE% 'Max. spaces.

      END IF

'Total record length including carriage return (CR) and line feed (LF).

      TOTLEN% = MAXSPC% + 2

      DAT$ = SPACE$(TOTLEN% + 11)  'For some reason 11 is a "magic number"

      TRULEN% = 0                  'Initialize length of data returned by device

      ZDAT$ = ""                   'Final destination of data string(s)

WTDAT: WHILE LEN(ZDAT$) < TOTLEN%

         CALL IOENTERS(HPADD&, DAT$, TOTLEN%, TRULEN%)    'Fetch the data

         ZDAT$ = ZDAT$ + LEFT$(DAT$, TRULEN%)    '2 bytes = empty string CR LF

      WEND

      FOR K% = 1 TO NDIODE%                     'Loop across all diodes monitored.

         MSB% = HEADER% + K% * BPRD%            'Most significant byte (MSB%)

         LSB% = MSB% - 2                        'Least significant byte (LSB%)

         N = ASC(MID$(ZDAT$, MSB%, 1))          'Decode data according to

         A$ = MID$(ZDAT$, LSB%, 2) + ZEROS$     'manual specifications.

         F1 = CSNG(CVL(A$))

         IF N <> 128 THEN

            IF N < 128 THEN

               A = N + (F1 / 65536)

            ELSEIF N > 128 THEN

               A = N - 256 + (F1 / 65536)

            END IF

         ELSE

```

```

            PRINT "BAD DATA DETECTED - SET TO ZERO."

            A = 0

         END IF

         YDATA(K%) = A           'Assign value to YDATA() array.

         IF VRNFLG% <> 0 THEN GOSUB VRNCE

      NEXT K%

      GOTO SAVED      'Jump section dealing with reading of variance data.

VRNCE:ZEROS$=CHR$(0) + CHR$(0) + CHR$(0)          'CVL needs 4 bytes.

                                   'Save and read variance values for every diode.

      VRNMSB% = HEADER% + K% * (BPRD% + 2)        'Most significant byte (MSB%)

      VRNLSB% = VRNMSB% - 1                       'Least significant byte (LSB%)

      M = ASC(MID$(ZDAT$, VRNMSB%, 1))            'Decode data according to

      VRN$ = MID$(ZDAT$, VRNLSB%, 1) + ZEROS$     'manual specifications.

      E1 = CSNG(CVL(VRN$))

      IF E1 <> 128 THEN

         IF E1 < 128 THEN

            VARIANCE = (M / 256) * 2 ^ E1

         ELSEIF E1 > 128 THEN

            VARIANCE = (M / 256) * 2 ^ (E1 - 256)

         END IF

      ELSE

         PRINT "BAD DATA DETECTED - SET TO ZERO."

         VARIANCE = 0

      END IF

      YVAR(K%) = VARIANCE              'Store value in YVAR() array.

      RETURN

SAVED:    'Save data as array or as single value if multiple or single diode monitored.

      IF DIODFLG% <> 0 THEN

         CNTCHK% = CNTCHK% + 1

         'Save data, point by point.

         CALL FLSAVE(YDATA(), YVAR(), NDIODE%, DIODFLG%, MINWV%, MAXWV%, WV1%, CNTCHK%)

      ELSE

         YDATA(1) = A

         CNTCHK% = CNTCHK% + 1

      END IF

END SUB

SUB HPGET (RESP$)
'This subroutine returns a string from the spectrophotometer through the
'HPIB interface using the IOENTERS driver subroutine.  RESP$ is the string
'returned, adjusted for its actual length.  If an error is detected, it is
'reported, but execution continues.  The variables ISC& (select code) and
'HPADD& (diode array device address) are assumed to be in shared common.
'======================================================================
        RSP$ = SPACE$(255)
        DO
           CALL IOENTERS(HPADD&, RSP$, 255, TRULEN%)
        'Check for error.
           IF PCIB.ERR <> NOERR THEN PRINT "Read Error - retrying."
        LOOP WHILE PCIB.ERR <> NOERR
        RSPLEN% = TRULEN% - 2
        IF RSPLEN% < 0 THEN RSPLEN% = 0
        RESP$ = LEFT$(RSP$, RSPLEN%)
END SUB

SUB HPINIT (NLIST%, WAVE%())
'Subroutine to initialize the diode array spectrophotometer.  It is
'responsible for the initialization sequence of the Hewlett-Packard
'diode array spectrophotometer. It checks the identity of the device,
'its calibration setting, lamp status, and other factors.  It also
'obtains from the user the analytical wavelength.  If the initialization
'sequence is not successful, the user is given the chance to make
'adjustments before the sequence is retried.
'The following variables in shared common are used:
'   WV1%    - analytical wavelength in nm.
'   MINWV% - minimum wavelength in nm
'   MAXWV% - maximum wavelength in nm
'   ISC&    - HPIB select code for diode array
'   HPADD& - HPIB device address for diode array
'   HPSTAT%- status of diode array (0=not initialized, 1=initialized,
'            2=initialized and reference run)
'=====================================================================
      TMOUT = 2              'Set time out to 2 seconds. Abort after 2 sec.
      CNTCHK% = -1           'Counter.
```

```
HPIBEG:
       PRINT: PRINT "About to open diode array."
       PRINT "Make sure it is ready and then ";
       CALL WTKEY
'First clear the device and the board.
       CALL IORESET(ISC&)
       CALL IOTIMEOUT(ISC&, TMOUT)
       CALL IOCLEAR(HPADD&)
'Now get device to identify itself and check if it's the right one.
       CALL HPSEND("IDY")
       CALL HPGET(IDY$)
       IF IDY$ <> "HP8452A,REV.A " THEN GOTO ERRFND
       CALL HPSEND("CAL")
       CALL HPGET(CAL$)
       ERRCHK% = VAL(CAL$) AND 3
       IF ERRCHK% <> 3 THEN GOTO ERRFND
'Set initial parameters for measurements.
                                  'Initialize all measurement parameters
       CALL HPSEND("DFL")         'to their defaults.
       CALL HPSEND("VRNO")        'Variance info. not calculated.
       CALL HPSEND("CCK")         'Clear frame counter clock.
       CALL HPSEND("SHU1")        'Keep shutter open during measurements.
       CALL HPSEND("LPS")         'Check current status of deuterium lamp.
       CALL HPGET(LPS$)           'Check if lamp off (0), on (1) or instrument
       ERRCHK% = VAL(LPS$)        'cover is currently off and so is lamp (2).
       IF ERRCHK% = 0 THEN        'Do what is necessary.
          PRINT "Turning on lamp - please wait."
          CALL HPSEND("LMP1")
          CALL PAUSE(60)
       END IF
       CALL HPSEND("LPS")
       CALL HPGET(LPS$)
       ERRCHK% = VAL(LPS$)
       IF ERRCHK% = 0 THEN GOTO ERRFND
       CALL HPSEND("GAJ")
       CALL PAUSE(5)
       PRINT "Diode array initialized."
       HPSTAT% = 1
'Ready to  start detector and make measurements.
       CALL DETSTRT(DIODFLG%, WV1%, MINWV%, MAXWV%, CNTCHK%, NLIST%, WAVE%())
       CALL GETAVG(DIODFLG%, WV1%, MINWV%, MAXWV%, CNTCHK%, NLIST%)
       EXIT SUB
ERRFND:          'Error detected - allow operator to make adjustments and try again.
        PRINT : PRINT "ERROR encountered in initialization of HP diode array."
        PRINT "Make necessary adjustments before continuing."
        GOTO HPIBEG
END SUB

SUB HPSEND (CMD$)
'This subroutine sends a string to the diode array using the HPIB driver
'subroutine IOOUTPUTS.  If an error is detected, a message is printed, but
'execution continues. The variables ISC& (select code) and HPADD& (diode
'array device address) are assumed to be in shared common.
'=====================================================================
      MES$ = CMD$
      CMDLEN% = LEN(MES$)
      DO
         CALL IOOUTPUTS(HPADD&, MES$, CMDLEN%) 'Subroutine found in FIDO library.
         IF PCIB.ERR <> NOERR THEN PRINT "Error on writing "; CMD$; " - retrying."
      LOOP WHILE PCIB.ERR <> NOERR
END SUB

SUB PARINIT (NLIST%, WAVE%())
'Subroutine to specify parameters such as wavelength(s), integration times,
'acquisition rates, and experimental conditions such as:
'single, list, or range of wavelengths
'     DIODFLG% = 0  flag for single wavelength
'     DIODFLG% = 1  flag for multiple wavelengths
'     DIODFLG% = 2  flag for list of wavelengths
'NLIST% is returned to calling program.  It is # of individual wavelengths.
'variance information
'     VRNFLG% = 0  flag for no variance calculation
'     VRNFLG% = 1  flag to calculate variance
```

```

'==========================================================================

SHUT:          'Want to keep shutter open during measurements ?

     PRINT "Enter:   <0> to open and close shutter or"

     PRINT "         <1> to keep shutter open during measurements"

     INPUT shutopt% : IF shutopt% <> 0 AND shutopt% <> 1 THEN GOTO SHUT

VARNCE:        'Wish to calculate variance of data?

     INPUT "Do you wish to calculate variance data <N>"; YN$

     YN$ = UCASE$(YN$)

     IF YN$ <> "Y" THEN

        VRNFLG% = 0

     ELSE

        VRNFLG% = 1

     END IF

ASKDIOD:       'Get the analytical wavelength

      CLS

      PRINT "Enter: <R> to work with wavelength Range."

      PRINT "       <S> to work with Single wavelength."

      PRINT "       <L> to work with a list of wavelengths."

      DO

         YN$ = INKEY$ : YN$ = UCASE$(YN$)

      LOOP WHILE YN$ = ""

     SELECT CASE YN$

        CASE "R"

           DIODFLG% = 1                   'Flag for multiwavelength data

GETWV1:    INPUT "Enter minimum wavelength (190 - 818) ", TEMP1%

           IF TEMP1% < 190 OR TEMP1% > 818 THEN GOTO GETWV1

           MINWV% = CINT(CSNG(TEMP1%) / 2) * 2

           IF MINWV% <> TEMP1% THEN

              PRINT "Wavelength resolution is 2 nm.!"

              PRINT "Actual wavelength used ="; MINWV%; " nm."

           END IF

GETWV2:    INPUT "Enter maximum wavelength ", TEMP2%

           IF TEMP2% < MINWV% OR TEMP2%   820 THEN

              PRINT "Max. wavelength must be > "; MINWV%; " and <= 820 !"

              GOTO GETWV2

           END IF

           MAXWV% = CINT(CSNG(TEMP2%) / 2) * 2

           IF MAXWV% <> TEMP2% THEN

              PRINT "Wavelength resolution is 2 nm.!"

              PRINT "Actual wavelength used ="; MAXWV%; " nm."

           END IF

           NDIODE% = 1 + (MAXWV% - MINWV%) / 2          'No. of diodes

        CASE "L"

           DIM WAVE%(20)                  'Array for discrete diodes.

           DIODFLG% = 2                   'Flag for list of wavelengths.

           INPUT "Enter number of discrete wavelengths to use <MAX. 20> "; NLIST%

           FOR I% = 1 TO NLIST%

              PRINT "Enter wavelength # "; I%;

              INPUT WAVE%(I%)

              WAVE1% = CINT(CSNG(WAVE%(I%)) / 2) * 2

              IF WAVE1% <> WAVE%(I%) THEN

                 WAVE%(I%) = WAVE1%

                 PRINT "Wavelength resolution is 2 nm.!"

                 PRINT "Actual wavelength used ="; WAVE1%; " nm."

              END IF

           NEXT I%

        CASE "S"

           DIODFLG% = 0                   'Flag for single wavelength

GETWV:     INPUT "Enter wavelength to use (190 - 820 nm.)"; TEMP%

           IF TEMP% < 190 OR TEMP% > 820 THEN GOTO GETWV

           WV1% = CINT(CSNG(TEMP%) / 2) * 2

           IF WV1% <> TEMP% THEN

              PRINT "Wavelength resolution is 2 nm.!"

              PRINT "Actual wavelength used ="; WV1%; " nm."

           END IF

        CASE ELSE

           BEEP

           GOTO ASKDIOD

     END SELECT

'Set defaults of integration time and sampling time intervals

'according to the HP specifications.

     IF NDIODE% > 35 THEN

        IF VRNFLG% = 1 THEN

           TEMPTIM = 1.8            'Set minimum sampling interval

        ELSEIF VRNFLG% = 0 THEN     'according to HP-8452A specifications.

           TEMPTIM = .6

```

```
         END IF
      ELSE
         TEMPTIM = .1
      END IF
'Obtain other parameters; integration time, sample interval,
INTVL: PRINT "Enter sampling interval for measurements ";
      PRINT "( "; TEMPTIM; " to 99999.9) ";
      PRINT "< "; TEMPTIM; " > ";
      INPUT SMINT
      IF SMINT < TEMPTIM OR SMINT > 99999.9 THEN GOTO INTVL
INTG: PRINT "Enter integration time (0.1 to "; SMINT; ") <0.1>";
      INPUT INTGTM
      IF INTGTM < .1 OR INTGTM > 25.5 THEN
         PRINT "Value must lie between 0.1 and "; INTGTM
         GOTO INTG
      ELSEIF INTGTM > SMINT THEN
         PRINT "Integration time cannot be larger than acquisition rate."
         GOTO INTG
      END IF
      IF shutopt% = 0 THEN
         IF (SMINT - INTGTM) < .5 THEN
            PRINT "<interval> must be 0.5 s greater than <integration>"
            GOTO INTVL
         END IF
      END IF
'Open file in readiness to receive data.
      INPUT "Enter file name to store data <+.EXT> ", FLNM$
      OPEN FLNM$ FOR OUTPUT AS #1
END SUB

SUB PAUSE (DLYTIM)

'Subroutine to wait a specified delay time (DLYTIM).

               TSTRT = TIMER

               DO

                  TNOW = TIMER

                  IF TNOW < TSTRT THEN TNOW = TNOW + 86400

                  TDIFF = TNOW - TSTRT

               LOOP UNTIL TDIFF >= DLYTIM

               END SUB

SUB WTKEY

'Subroutine to wait for user to strike a key.

        PRINT "Hit any key to continue."

CLR1:   A$ = INKEY$                         'Clear the keyboard

        IF LEN(A$) <> 0 THEN GOTO CLR1      'buffer of spurious inputs

WT1:    A$ = INKEY$                         'Now wait for a key to be struck

        IF LEN(A$) = 0 THEN GOTO WT1

END SUB

